# Lung Expansion Therapy for the Potential Populations: A Literature Review

**DOI:** 10.7759/cureus.49283

**Published:** 2023-11-23

**Authors:** A. Sankarganesh, Kumaresan Abathsagayam, Natesh Prabu Ravisankar, Nityal Kumar Alagingi

**Affiliations:** 1 Department of Physiotherapy, Saveetha College of Physiotherapy, Saveetha Institute of Medical and Technical Sciences (SIMATS), Chennai, IND; 2 Department of Physiotherapy, St John's Medical College Hospital, Bangalore, IND; 3 Department of Critical Care Medicine, St John's Medical College Hospital, Bangalore, IND; 4 Department of Physiotherapy, Nitte Institute of Physiotherapy, NITTE (Deemed to be University), Mangalore, IND

**Keywords:** balloon-blowing exercise, postoperative pulmonary complications, tracheostomy, lung expansion therapy, incentive spirometer

## Abstract

Secretion retention, atelectasis, and reduced lung compliance occur when endotracheal or tracheostomised patients cough ineffectively. There is a possibility of infection if the collapsed regions of the lungs are not reinflated. Therefore, to improve clinical outcomes, such as diaphragm mobility and thickness, lung volume, and thickness of the abdominal muscles, and decrease the length of hospitalizations, mechanical and manual techniques, such as balloon blowing exercises and incentive spirometer, are required. PubMed, Google Scholar, Pedro, Clinical Keys, Helinet, ProQuest, and Science Direct databases were used for the literature search considering the inclusion and exclusion criteria. The several manual and mechanical methods that were employed for lung expansion treatment for the potential populations are highlighted in this overview of the literature. Ten studies were considered in this review: five on balloon-blowing exercises, four on tracheostomy with incentive spirometry exercises, and one on incentive spirometry with balloon-blowing exercises. The effects were examined on individual outcomes that included rate of perceived exertion (RPE), diaphragm mobility, pulmonary function, volume of breath, length of hospitalization, and postoperative day complications. The structured protocols proved to be effective in improving lung expansion and pulmonary function for the potential population that involved healthy adults, noncritical COVID-19 adults, smokers, thoracotomy patients, and tracheostomised patients. The road to recovery is yet unexplored and underachieved because of the lack of evidence.

## Introduction and background

The incentive spirometer, as first introduced by Robert H. Barlett in 1973, is a tool that promotes consistent, sustained maximal inspiration through visual feedback. Because of its low risk and high efficacy, it is being utilized in the majority of hospitals nationwide because of its affordability, simplicity of usage, and safety profile. According to surveys, 71% to 95% of hospitals that perform abdominal and cardiothoracic procedures employ it [1,2].

Retention of secretions, reduced lung compliance, and atelectasis can result from ineffective coughing. If the collapsed lung parts are not reinflated, they may get infected. The technique that expands the lung beyond the capacity of regular unassisted inspiration is known as a lung expansion technique [3]. In patients who are unable or unwilling to undertake periodic hyperinflation, such as those who have undergone thoracic or abdominal surgery, have obstructive pulmonary illness, or have neuromuscular or chest wall abnormalities, lung expansion procedures are recommended as a preventative measure against atelectasis or pneumonia [4].

Locoregional and microvascular free tissue transfers used in head and neck surgery have been shown to be associated with an increased risk of pulmonary complications [5]. Extended duration of general anesthesia, coupled with the use of narcotic analgesics during the postoperative phase, results in reduced efficacy of coughing, impaired mucociliary clearance, and pulmonary secretion retention, all of which exacerbate the risk of atelectasis and pneumonia [6].

Incentive spirometry should be offered to any patient who requires chest physiotherapy because of an in-situ tracheostomy tube that can be customized as demonstrated by Goldstein et al. in their study. The components of the customized spirometer included AirLife incentive spirometer (Cardinal Health, Dublin, Ohio), bacterial/viral filter, two sets of gooseneck tubing, wye adapter, and Shiley tracheostomy tube (Covidien-Nellcor, Boulder, Colorado). The standard tracheostomy tube with an outer diameter of 12.0 mm is connected to the incentive spirometer arrangement. The wye adapter can be used to give extra oxygen or to enable breathing for the patient while the device is connected. It is also simple to occlude the wye adapter's extra arm, enabling direct communication with the incentive spirometer [7]. As a result, this can lead patients to be active in their own healing and avoid lung-related complications. With an incentive spirometry device, patients can be visually motivated to take slow, deep breaths, which are followed by holding their breaths. Incentive spirometers are lightweight and portable, and they are divided into one of two categories: volume-oriented devices (ISVOD) or flow-oriented devices (ISFOD) [8]. Although incentive spirometry is widely used, several systematic reviews have not found strong evidence supporting its usefulness in avoiding postoperative problems [9]. Hence, this review analyzed the effect of balloon-blowing exercises (BBEs) on healthy adults, noncritical adult COVID-19 patients, and smokers. The review also analyzed the effects of incentive spirometry and BBEs on thoracotomy patients. Furthermore, the impact of the incentive spirometer along with the modifications made to it for tracheostomised patients was also reviewed in this review of the literature.

## Review

Search methodology

**Figure 1:**
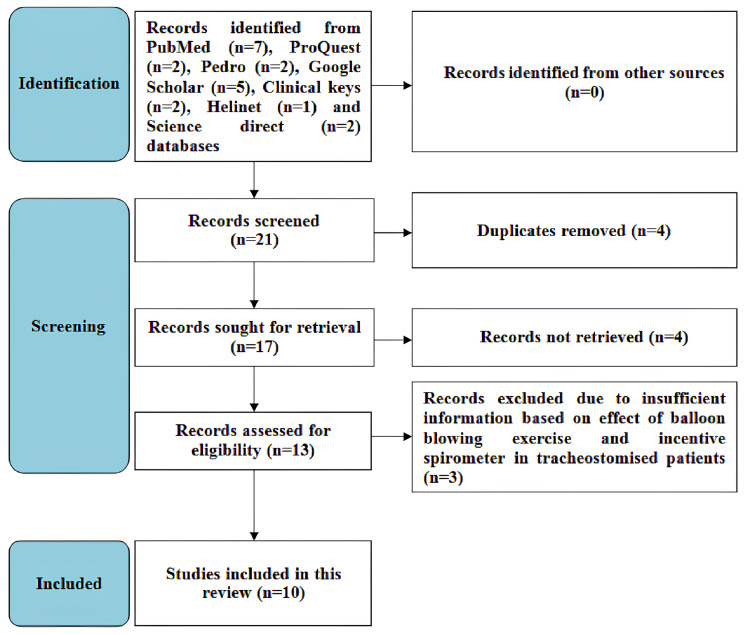
Search strategy flowchart

PubMed, Google Scholar, Pedro, Clinical Keys, ProQuest, Helinet, and Science Direct databases were used for literature search, and articles that were published between 2007 and 2023 were included in this review. The keywords that were used to search the articles included “lung expansion therapy,” “incentive spirometer,” “balloon blowing exercise,” and “tracheostomy.” The inclusion criteria included full-text articles, English-language articles discussing the effects of balloon-blowing exercise (BBE) and incentive spirometers on tracheostomised patients, and studies involving only humans who were more than or equal to 18 years old. Studies conducted on animals, articles not published in English, and articles not providing a brief description of the effects of BBE and incentive spirometer in tracheostomised patients were excluded from the analysis. Ten articles in all were included in this review after considering the inclusion and exclusion criteria. The search strategy employed in this review adhered to the Preferred Reporting Items for Systematic Reviews and Meta-Analysis (PRISMA) criteria (Figure 1).

Data extraction

Table 1 provides a brief description of the data, which includes the author, sample size, population, methodology, outcome measures, and conclusion of the findings included in this review.

**Table 1:** A literature review of the studies RCT = Randomised controlled trial, BBE = Balloon blowing exercise, RBS = Rendell-Baker-Soucek pediatric facemask, RPE = Rate of perceived exertion, PEF = Peak expiratory flow, PEFR = Peak expiratory flow rate, VC = Vital capacity, IRV = Inspiratory reserve volume, ERV = Expiratory reserve volume, FEV1 = Forced expiratory volume exhaled in the first second, FEV1/FVC = Forced expiratory volume exhaled in the first second divided by the forced vital capacity

Author	Sample size (n)	Study type	Population	Methods/suggestions	Outcome measures	Conclusion
Studies related to BBE						
Mohammad Bargahi et al. [10]	80	RCT	≥18 years	BBE group (n=40) and control group (n=40).	SpO2, Modified Borg Dyspnea Scale	In noncritical COVID-19 patients, BBE does not appreciably improve oxygenation but improves dyspnoea.
Seo et al. [11]	20	RCT	21-23 Years	The control group involved diaphragmatic exercise, whereas the experimental group performed BBE.	PEF, FEV, FVC, and VC were measured at one second.	To increase lung function BBE using a ball in a 90/90 bridge position can be performed.
Lee et al. [12]	21	RCT	23-26 years	Group I performed abdominal exercises, group II performed BBE, and group III performed both.	PEFR	Abdominal muscle strength and breathing capacity may be significantly impacted by upper abdominal exercises and BBE.
Kim et al. [13]	30	RCT	22-25 years	The control group did not perform any exercise, whereas the experimental group involved BBE three times a week for eight weeks. Both groups consisted of 15 participants each.	PEF, ERV, VC IRV, FEV1, FVC FEV1/FVC	The lung function improved with the implication of BBE.
Kanniappan et al. [14]	100	Quasi-experimental study	25 to 50 years	Group A performed breathing exercises, and Group B performed BBE.	PEFR	According to the study's findings, Group B's posttest PEFR significantly increased.
Studies related to the comparison of BBE with incentive spirometry						
Ghani et al. [15]	48	RCT	41-63 years	Participants were divided into two groups of 24 each. The control group involved incentive spirometry along with conventional respiratory physiotherapy whereas, the experimental group performed BBE in combination with conventional respiratory physiotherapy.	Tape measure, digital spirometer, RPE scale, and pedometer	For thoracotomy patients undergoing lung surgery, BBE combined with conventional/routine respiratory physiotherapy can serve as a substitute for incentive spirometers in preventing post-operative pulmonary problems or in producing similar results as that incentive spirometer.
Studies related to incentive spirometry for tracheostomised patients						
Goldstein et al. [7]	10	Prospective case series	41-63 years	With the tracheostomy cuff inflated, patients performed prolonged maximal inspiration exercises using the incentive spirometry equipment. Up to ten cycles of exercises were performed during waking hours, while the patient was sitting up in bed or a chair until ambulation was possible.	The volume of breath, time to ambulation, length of hospitalization, and postoperative day complication.	The usage of an incentive spirometer that was adapted for tracheostomy patients is described, which also confirms its feasibility of using this device safely as it was observed in 10 patients.
Bloria et al. [16]		Clinical suggestions		It is possible to modify the incentive spirometer for tracheostomized patients with motor neuron disease, head and neck surgical patients, and patients with cervical spine damage.		The modification in incentive spirometer was not tested in metallic tracheostomy tubes, and, additionally, determination of the impact of incentive spirometry on patient's overall outcomes is also necessary.
Garg et al. [17]		Clinical suggestions		RBS pediatric facemask, a straight connector or adaptor, and an incentive spirometer were the tools utilized. Anesthesia tubing connectors form the adapter. It is fastened to the RBS pediatric facemask on one side and the incentive spirometer's tubing on the other. The stoma is covered with an RBS mask of appropriate size. A cotton pad with a central hole can also be used for complete sealing. The patient securely covers the stoma with this assembly mask, inhales through the spirometer, and is instructed to maintain maximal inspiration for around three seconds before expiration after removing the RBS mask.		To facilitate lung expansion in patients with tracheostomy stomas without requiring the insertion of a tracheostomy tube, modified spirometer assemblies may prove to be an effective tool.
Malhotra et al. [18]		Clinical suggestions		Patients often place the mouthpiece in their mouths and breathe through it. Nevertheless, the incentive spirometer needs to be modified to be used with patients who have tracheostomies. In this study, the incentive spirometer was successfully modified, which is readily accessible, low-cost technology, and the patients started utilizing it as a lung expansion technique on tracheostomised patients admitted to the intensive care unit.		In summary, incentive spirometry is more effective when combined with chest physical therapy, early mobilization, and adequate analgesia.

Discussion

This literature review highlights various manual and mechanical techniques used for lung expansion for the potential populations. In total, 10 articles were included in this review of which four articles were about tracheostomy with incentive spirometry exercises, five consisting of BBE, and one of incentive spirometry with BBE. The effects of the techniques were examined on individual outcomes that involved diaphragm mobility, RPE, pulmonary function, length of hospitalization, volume of breath, and postoperative day complications. The results of the studies demonstrated that both BBE and incentive spirometry are effective in improving lung function when combined with chest physical therapy, early mobilization, and adequate analgesia. Additionally, BBE can be used as an alternative for incentive spirometry. Furthermore, in patients with tracheostomy stomas, modified spirometer assemblies may prove to be an effective tool without requiring the insertion of a tracheostomy tube.

Incentive spirometry should be made available to every patient in need of chest physical therapy who has an in situ tracheostomy tube. This will enhance diaphragm mobility, enable patients to take an active role in their own recovery, and reduce pulmonary problems. A prospective case series on incentive spirometry for tracheostomy patients was conducted by Goldstein et al. on a total of 10 patients wherein the mean duration of anesthesia was nine hours, 70% of patients had microvascular free flap reconstruction, and 60% were current or former tobacco users. During the postoperative period, patients utilized the incentive spirometer for a mean of 1.6 days, averaging 3.3 sessions per day and 6.8 breaths per session. Patients responded positively to the device, and there were no side effects from using it. This study creates a safety profile for the device to be used in future research and at last supports the viability of applying a customized incentive spirometer for tracheostomy patients. Suggested the directions for the future, research concentrating on the investigation of the outcomes is necessary, although the use of incentive spirometry in tracheostomy patients shows promise for achieving similar outcomes as its use in non-tracheostomy patients [7].

In their clinical recommendation on incentive spirometry for tracheostomised patients, Malhotra et al. showed that patients typically place the mouthpiece in their mouths and use the incentive spirometer to inspire. However, the incentive spirometer needs to be modified for patients who have tracheostomies. These simple, affordable, and readily available devices have been effectively utilized for the lung expansion technique on tracheostomised patients admitted to the intensive care unit [18].

An RCT by Lee et al. examined the effects of BBE and upper abdominal training on respiratory rehabilitation. The researchers concluded that these activities may significantly affect respiratory capacity and abdominal muscle strength [12]. In an RCT, Seo et al. found that BBE, performed with a balloon in a 90/90 bridge position, can be used to enhance pulmonary function [11]. Similarly, Kim et al. reported that after eight weeks of performing the prescribed BBE three times a week, the outcomes demonstrated that the exercise improved lung function [13].

An RCT was conducted by Bargahi et al. to determine the effect of BBE on oxygenation and dyspnea in adult COVID-19 patients who were not critically ill. Eighty patients were randomized to a breathing exercise intervention group and a control group consisting of 40 patients each. In addition to receiving various therapies, the participants in the intervention group were asked to lie down and blow a balloon five times a day. The dyspnea of the BBE group significantly improved, according to the data [10]. Boyle et al. suggested that during neuromuscular training and a range of stabilizing movements, the BBE is a specific example of an exercise that could be helpful for integrating the co-activation of deep abdominal muscles with the pelvic floor and diaphragm [19]. The BBE is a conservative exercise designed to help patients achieve diaphragm, spinal position, and neuromotor control (lumbar-pelvic stability) for optimal posture and breathing. Nevertheless, no research or experimental testing has been done on the BBE for tracheostomized individuals, either with or without incentive spirometry.

An RCT with a sample size of 48 subjects, 24 in the control group in which incentive spirometry along with conventional chest physiotherapy was performed and 24 in the experimental group who underwent conventional chest physiotherapy along with BBE. Data were gathered at baseline, three days, and five days following exercise therapy, and it was determined that BBE can be performed as an alternative to incentive spirometer in thoracotomy patients who underwent lung surgeries, either to overcome postoperative pulmonary complications or to achieve the same results as incentive spirometer in conjunction with conventional respiratory physiotherapy [15]. In a prior investigation, diaphragmatic mobility was assessed using ultrasound while incentive spirometers were being used. The findings demonstrated that, in comparison to ISFOD, diaphragmatic mobility was much greater during ISVOD and diaphragmatic breathing (DB) [20].

All these studies did not consider the effect of incentive spirometry and BBE on improving diaphragm mobility among tracheostomy patients. Hence, more experimental studies to confirm the effectiveness of volume-oriented incentive spirometry on diaphragmatic mobility in patients with tracheostomy are required. Additionally, future research can be conducted with specific age groups that can involve young and old populations separately, on different conditions of cardiopulmonary patients along with long follow-up.

## Conclusions

The articles reviewed in this study showed evidence that BBE was effective in improving lung function, dyspnoea, abdominal muscle strength, peak expiratory flow rate, and breathing capacity. Additionally, BBE can be used as an alternative to incentive spirometry in patients who underwent lung surgeries, either to overcome postoperative pulmonary complications or to achieve the same results as incentive spirometer in conjunction with conventional respiratory physiotherapy. Similarly, customized incentive spirometry improved lung function in the pulmonary rehabilitation program for tracheostomy patients. The structured protocols have been proven to be effective in improving lung expansion and pulmonary function for potential populations that involved healthy adults, noncritical COVID-19 adults, smokers, thoracotomy patients, and tracheostomised patients. Because of a lack of evidence, the road to recovery remains untouched and underachieved. This study emphasizes the need to undertake more intervention studies to establish a standard modified incentive spirometry device to adapt tracheostomy tubes to promote diaphragm mobility and lung expansion with real-time ultrasound for diaphragm length and/or changes in abdominal muscle thickness.
